# A Case Report of Unilateral Orolingual Angioedema Secondary to Alteplase Administration

**DOI:** 10.7759/cureus.2869

**Published:** 2018-06-23

**Authors:** Dustin Harris, David Harter

**Affiliations:** 1 Emergency Department, The University of Chicago Medicine, Chicago, USA

**Keywords:** angioedema, stroke, unilateral, tpa, alteplase, orolingual

## Abstract

Emergency physicians should be aware of adverse drug reactions prior to administering medication. Alteplase, or tissue plasminogen activator (tPA), is a common medication in the emergency department, whether it is being used for a stroke or pulmonary embolus. Angioedema can be caused by almost any medication. tPA administration can cause an atypical form of angioedema. The following case was one of unilateral orolingual angioedema associated with tPA administration in the emergency department in a stroke patient. The mechanism of tPA-induced angioedema is poorly understood. Angioedema can be treated with stopping the infusion of medication, Benadryl® (Johnson & Johnson Consumer, Inc., Fort Washington, PA), histamine antagonists, steroids, and epinephrine. Angioedema is a life-threatening event in certain situations, and emergency medicine providers would do well knowing how to approach these cases.

## Introduction

Adverse drug reactions may affect up to 20% of hospitalized patients and, worldwide, may be responsible for up to 20% of fatalities due to anaphylaxis [1]. A knowledge of allergy history is important information to all clinicians and can influence the approach to patient workup and treatment. A severe form of allergic reaction is angioedema, which is mediated by histamine or bradykinin release [2]. Angioedema results from the dilatation of the capillaries beneath the skin or mucosa, causing localized edema [3-4]. Angioedema occurring in the mouth (orolingual) or throat constitutes an airway emergency.

Although uncommon, orolingual angioedema has been associated with the administration of tPA [5]. Alteplase has been shown to cause angioedema in about 1.3% to 5% of cases [3, 5]. Factors associated with an increased risk of angioedema secondary to tPA administration include concurrent angiotensin-converting enzyme (ACE) inhibitor use and insular infarcts [5]. Ten percent of patients with insular infarcts and up to 17% of patients on ACE inhibitors are susceptible to orolingual angioedema after tPA [5]. The case below depicts the management of a stroke patient who experienced unilateral orolingual angioedema in the emergency department after tPA administration.

## Case presentation

A 71-year-old African American female with a past medical history of hypertension and cerebrovascular accident in 2004 presented to the University of Chicago emergency department with sudden onset dysarthria. The patient had noted a similar episode three days prior that spontaneously resolved. The patient’s speech became slurred 10 minutes prior to her arrival in the emergency department. Her home medications included daily aspirin, diltiazem, and lisinopril. She had been using Lisinopril for two years without difficulty and had taken her last dose on the previous morning. Further intake history was limited secondary to dysarthria, causing the patient to stutter and have difficulty with word finding. Vital signs included a blood pressure of 163 mmHg/83 mmHg, a pulse of 67 beats per minute, respiratory rate of 17 breaths per minute, and oxygen saturation of 97% on room air. On physical exam, we found the patient to have a right-sided facial droop and 4/5 motor strength in the right arm/leg compared to 5/5 in the left arm/leg, as well as tongue deviation to the right and an initial NIH Stroke Scale Score of 4. A brain computed tomography (CT) scan was unremarkable for acute intracranial hemorrhage or ischemic stroke but did demonstrate an area of encephalomalacia in the left cerebellum and the frontal and periventricular white matter. After neurologist and pharmacy input, the decision was made to administer tPA based on concern for acute ischemic stroke symptoms. The patient was consented for treatment, and tPA, 0.09 mg/kg followed by 0.81 mg/kg (patient weighed 70.7 kg), was administered intravenously (IV) one hour after patient arrival.

**Figure 1:**
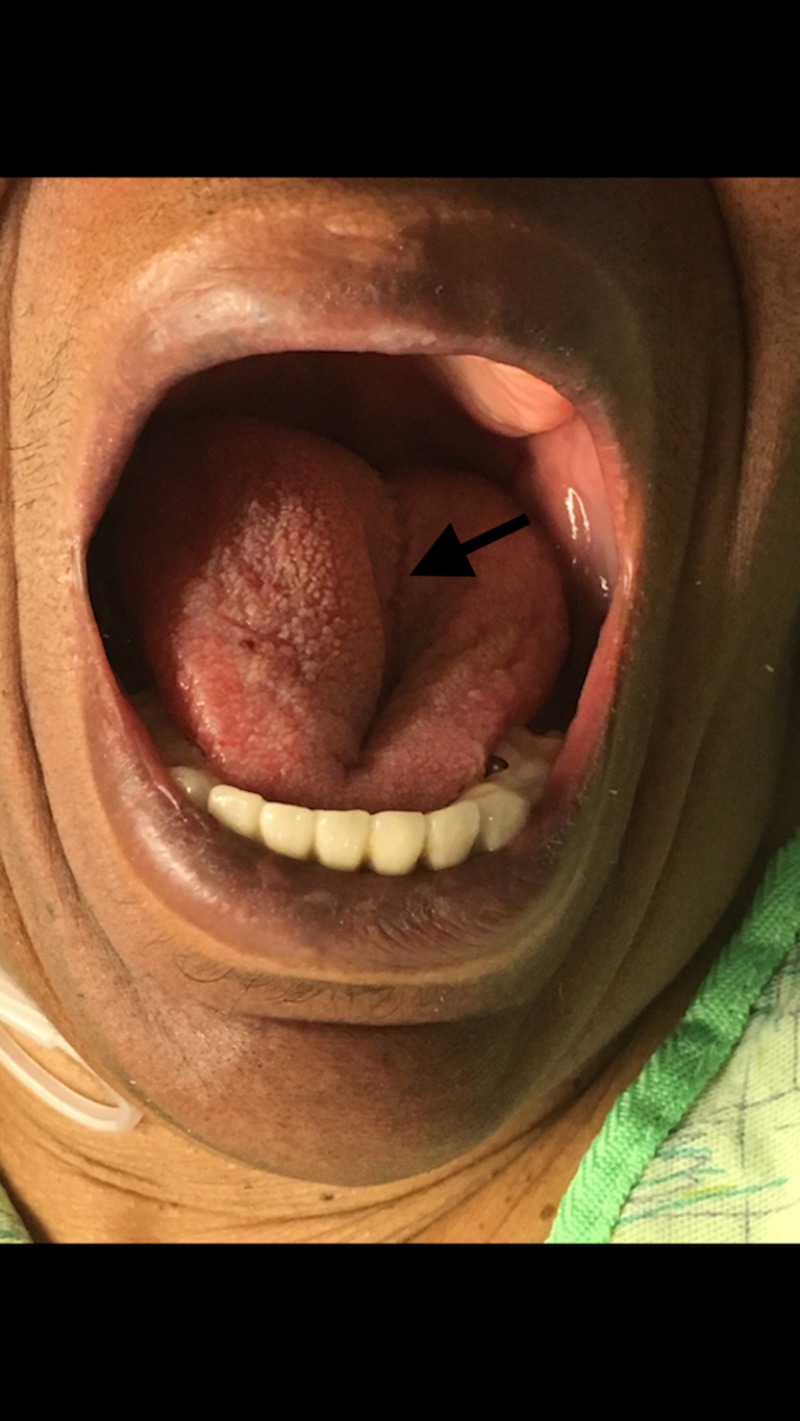
Right-sided tongue edema

The patient’s symptoms improved 10 minutes after tPA administration. Within 30 minutes, the patient’s tongue developed a 1-centimeter maroon area of swelling on the right side that appeared to be consistent with a small hematoma. It was thought that the patient may have bitten her tongue and a hematoma was expanding secondary to the tPA. Over the course of 20 minutes, the lesion continued to expand until it reached maximum size seen below (Figure 1). The swelling of the tongue remained unilateral and there was no adjacent swelling or urticarial rash of the lips or face. An otolaryngologist was consulted to perform a bedside laryngoscopy to assess the patient’s airway. The otolaryngologist noted swelling in the oropharynx with a patent airway. The patient was given diphenhydramine, methylprednisolone, and famotidine and was admitted to the neurology unit. Her C1 esterase level was normal. She recovered over the next 24 hours and was discharged from the hospital on hospital day 4 with instructions to continue her home medications, including the Lisinopril, and with the addition of Keppra® (UCB Pharma, Brussels, Belgium).

## Discussion

A literature review demonstrated an overall small number of cases of unilateral angioedema secondary to tPA administration. This particular case had some similar characteristics to those cases [3-4]. The patient had stroke-like symptoms, but all imaging was negative for acute signs of an ischemic stroke. The angioedema did coincide with the side of her symptoms and she was, at the time, taking a daily ACE inhibitor. The mechanism of angioedema after tPA is not completely understood. Much like angioedema experienced while taking an ACE inhibitor, it is thought that tPA causes a buildup of bradykinin by converting plasminogen to plasmin and subsequently causing a systemic release of histamine by mast cells [3-4]. One study noted a significant correlation with the location of the ischemic brain lesion and the side of the angioedema but did not note a correlation with the side of symptoms [6].

If one does encounter a patient experiencing angioedema during or after tPA administration, the University of Cincinnati has developed a protocol for the next steps in management. It involves a stepwise approach similar to management of other cases of an allergic reaction involving: stopping the infusion, diphenhydramine, histamine antagonists, steroids, epinephrine, and an otolaryngology/anesthesia consult if needed [7]. The patient’s airway should be assessed every 15 - 20 minutes prior, during, and after tPA administration and any changes treated accordingly [7-8].

## Conclusions

We believe angioedema should also be considered during tPA administration as it could have life-threatening effects. If the patient has dyspnea, swallowing difficulty, or noted swelling, consider tPA-associated angioedema in the differential diagnosis and apply the treatment strategy noted above.
